# Research on daily medication and drug efficacy evaluation for chronic diseases based on natural language processing (NLP)

**DOI:** 10.3389/fpubh.2026.1780308

**Published:** 2026-03-23

**Authors:** Tao Jiang, Zhini Yu, Liangliang Zhang, Liyuan Ge, Xue Wang, Tingting Li, Na Wang, Zhihua Wang

**Affiliations:** 1 School of Public Health, Guilin Medical University, Guilin, China; 2 School of Humanities and Management, Guilin Medical University, Guilin, China; 3 School of Basic Medical Sciences, Guilin Medical University, Guilin, China

**Keywords:** chronic disease management, daily medication, drug efficacy evaluation, hypertension drug Lisinopril, Natural Language Processing (NLP)

## Abstract

**Objectives:**

This study aims to harness Natural Language Processing (NLP) to improve chronic disease medication management, focusing on the antihypertensive drug Lisinopril. The objectives are: (1) to develop an intelligent drug safety monitoring and personalized intervention platform integrating patient feedback; (2) to accurately identify drug side effects and analyze their associations with patient demographics; and (3) to build a personalized medication recommendation system for enhanced clinical decision-making.

**Methods:**

We used user reviews from authoritative medical databases (WebMD and Kaggle) spanning 2008–2020, forming a high-quality dataset of 345,845 samples related to Lisinopril. Data preprocessing involved redundancy removal, missing value imputation (e.g., using the KNN algorithm), and text standardization (e.g., through UMLS terminology mapping). NLP techniques included sentiment analysis and entity recognition with BERT models, topic modeling using LDA algorithms to extract key side-effect patterns (e.g., dry cough, dizziness), as well as the integration of knowledge graphs and decision trees for personalized recommendations, and the development of an interactive risk heatmap dashboard for dynamic monitoring.

**Results:**

The analysis revealed that common side effects of Lisinopril included dry cough (38%), dizziness (28%), and fatigue (22%), with nearly half of users experiencing these issues—particularly among older adults and long-term users. Sentiment analysis showed that 58% of reviews were negative, primarily due to side effects. The personalized recommendation system, tested in simulated scenarios, significantly improved medication adherence by suggesting alternatives (e.g., ARBs for cough-sensitive patients). The NLP-driven framework achieved 89.7% accuracy in sentiment classification and identified side-effect patterns 15 times faster than manual annotation.

**Conclusion:**

NLP effectively extracts insights from patient feedback, enabling rapid identification of drug efficacy and side effects. The study demonstrates the potential of NLP to support chronic disease management and precision medicine by facilitating early risk detection and personalized interventions. Future work should address subjective bias in reviews and integrate multi-source data for comprehensive assessment models.

## 1 Introduction

Chronic disease management poses a perennial public health challenge globally (1). Conditions such as hypertension typically necessitate long-term, often lifelong pharmacotherapy, making the continuous evaluation of drug safety and efficacy essential (2). However, traditional pharmacovigilance methods face significant limitations (3). Clinical trials may not capture all potential risks prior to drug approval, and some serious side effects only emerge after widespread use. Meanwhile, supplementary approaches such as spontaneous reporting and surveys are often constrained by reporting delays and limited population coverage (4), which restricts a comprehensive understanding of drug safety profiles. These constraints collectively impede the timely detection of complex adverse reaction signals in real-world settings. At the same time, vast amounts of unstructured patient-generated data from electronic health records and online platforms remain underutilized due to the inefficiency of manual processing (5).

Recent advances in Natural Language Processing (NLP), including pre-trained language models, fine-grained sentiment analysis, and topic modeling, offer powerful tools for the automated extraction of insights from medical text (6). A growing body of research has demonstrated the value of NLP in pharmacovigilance and drug safety monitoring. Khemani et al. (7) proposed a hybrid AI-driven framework integrating structured (e.g., patient demographics, lab results) and unstructured data (e.g., clinical notes) to detect adverse drug reactions (ADRs), demonstrating that CNN models with BERT achieved superior accuracy in identifying potential ADRs across patient subgroups, outperforming traditional models such as Logistic Regression (78%) and Support Vector Machines (80%). Farooq et al. (8) leveraged digital media data for pharmacovigilance, collecting tweets for 11 different drugs and constructing machine learning models for automatically annotating publicly available Twitter data; their results showed high concordance with FAERS, Medeffect, and Drugs.com, and they were able to recover on average 7 known side effects from Twitter data that were not reported on FAERS. Xuan et al. (9) proposed MVDSA, a novel multi-view drug-side effect association prediction model that integrates multiple relationship semantics, local topologies of knowledge graphs, and multi-view features of drug-side effect entity pairs; extensive experiments demonstrated that MVDSA outperforms 10 state-of-the-art methods in predicting drug-side effect associations. Rudnisky et al. (10) conducted a comprehensive review of pharmacovigilance in the era of artificial intelligence, concluding that through ML algorithms, ADRs can be identified more quickly and accurately compared to traditional PV methods, while using NLP models, AI is able to extract relevant patient data from unstructured data sources such as electronic health records (EHRs) and report certain drug interactions more accurately and efficiently.

Despite these advances, existing research often focuses on optimizing models for individual data sources or isolated tasks. For example, while social media mining has proven valuable for signal detection (8), it typically lacks the ability to integrate with demographic and clinical characteristics. Similarly, electronic health record-based studies (7) provide clinically validated data but may not capture the nuanced patient experiences found in online forums. A significant gap remains: the absence of an integrated framework capable of synthesizing multi-source data and translating analytical insights into actionable clinical decision support. This is especially critical for enabling dynamic, personalized risk assessment and management in long-term medication safety for chronic diseases (11, 12).

To address this gap, this study develops a comprehensive NLP-based framework for intelligent medication safety monitoring in chronic care. We propose to systematically integrate multi-source patient-generated text. By employing techniques including Named Entity Recognition (NER), sentiment analysis, and topic modeling (11), this framework aims to accurately and automatically extract signals of adverse drug reactions and investigate their associations with patient demographics and clinical characteristics. Furthermore, we explore the feasibility of constructing personalized risk profiles based on analytical outcomes to inform clinical decision-making. Ultimately, this work aims to provide novel methodological support and practical tools to enhance the responsiveness and precision of pharmacovigilance systems, advancing the paradigm of personalized chronic disease management (13, 14).

## 2 Method

### 2.1 Data sources and preprocessing

The data for this study were sourced from the globally recognized healthcare platform WebMD, and were obtained via authorized data collection from the data science community Kaggle.^1^ The dataset comprises a total of 360,000 drug reviews spanning the period from 2008 to 2020. These reviews cover treatments for 12 common conditions, including hypertension, diabetes, and depression. For the present study, the analysis focuses specifically on reviews of the antihypertensive drug Lisinopril. The raw data contains the following fields (Table 1).

**Table 1:** Raw data fields.

Field	Example	Description
Medication	Lisinopril	Generic and brand names
Indication	High blood pressure	Medical condition, associated with ICD-10 codes (e.g., I10)
Adverse event	Persistent dry cough at night	Free-text description including symptom, severity, and duration
Patient age	45–54	Provided in pre-defined ranges (25–34, 35–44, …, 75+)
Patient gender	Female	Recorded as Male, Female, or Other
Effectiveness rating	1	5-point Likert scale (1 = Very poor, 5 = Excellent)
Satisfaction rating	5	5-point Likert scale (1 = Very dissatisfied, 5 = Very satisfied)

### 2.2 Data cleaning and standardization

#### 2.2.1 Handling of redundant data and missing values

*Duplicate removal*: A unique hash value (fingerprint) was generated for each patient review using the SHA-256 algorithm. Exact duplicates, such as those from repeated submissions by the same user, were identified and removed. A total of 12,403 duplicate records (3.4% of the dataset) were eliminated.

*Missing value imputation*: A hierarchical imputation strategy was applied. Records with missing values in critical fields (e.g., medication name, adverse event description) were excluded, accounting for the removal of 8,752 records (2.4%). For missing values in less critical fields (e.g., age, gender), the k-nearest neighbors (KNN) algorithm (*k* = 5) was used to impute values based on similar textual features. For example, if an adverse event description included phrases like “headache during pregnancy,” the missing gender field was inferred as “female.”

#### 2.2.2 Text standardization and feature enhancement

Stop-word Filtering: An extended medical stop-word list containing 1,200 terms was applied to remove common, low-information-content words (e.g., “I,” “the,” “patient”). Relevant medical abbreviations, units, and terminology (e.g., “mm Hg,” “q.d.”) were retained.

Stemming and Lemmatization: For English text processing, word stemming was carried out using the Snowball stemmer. For Chinese reviews, word segmentation and standardization were conducted using the LTP toolkit, which included simplifying synonymous symptom descriptions (e.g., reducing “dizziness and blurred vision” to the core symptom “dizziness”).

Symptom Terminology Mapping: Descriptive symptom phrases were mapped to standardized medical terminology using the UMLS (Unified Medical Language System) Metathesaurus. For instance, lay descriptions such as “coughing severely at night preventing sleep” were mapped to the standard clinical term “nocturnal dry cough” alongside its associated ICD-10 code (R05.3).

#### 2.2.3 Privacy protection and anonymization

To safeguard patient privacy and comply with ethical data use standards, the dataset underwent a multi-layered anonymization process.

*Direct identifier removal*: Direct personal identifiers were de-identified. User IDs were replaced with irreversible MD5 hash values (e.g., “User123” → “e10adc3949ba59abbe56e057f20f883e”). Geographic locations were generalized to broader administrative regions (e.g., “Manhattan, New York City” was generalized to “Northeastern United States”).

*Quasi-identifier generalization*: Quasi-identifiers were subjected to generalization or binning. Age was grouped into 10-year intervals (e.g., “58 years old” was generalized to “50–59 years”). The precision of timestamps was reduced to year and month only (e.g., “2020-05-15” became “2020-05”).

*Differential privacy reinforcement*: To provide a formal privacy guarantee and mitigate composition attacks, Laplacian noise (*λ* = 0.1) was added to textual field attributes. This ensures that the inclusion or exclusion of any single individual’s data has a negligible impact on the analytical outcomes.

*Characteristics of the processed dataset*: Following the cleaning and anonymization procedures, a high-quality, analysis-ready dataset of 345,845 valid records was obtained. Its key characteristics are summarized below:

*Field completeness*: A completeness rate of 99.6% was achieved for critical fields, including medication name, ratings, and adverse event descriptions.

*Text normalization efficacy*: The average review length was reduced from 187 to 63 words, increasing the information density by a factor of 2.97.

The dataset was validated to satisfy k-anonymity (*k* = 15) and l-diversity (l = 8) criteria. The estimated risk of successful re-identification was calculated to be below 0.1%.

This processed dataset provides a robust foundation for subsequent drug safety analysis, enabling the investigation of potential risk and benefit signals from a large corpus of real-world patient experiences.

### 2.3 Analytical framework

#### 2.3.1 Sentiment analysis

Formula for BERT-Based Positive–Negative Classification of Reviews: where (Si) denotes a single review, and n represents the total number of reviews:

$$\text{Sentiment Score}=\frac{\sum_{i=1}^{n}\text{BERT}(s_{i})}{n}$$

#### 2.3.2 Topic modeling: application of LDA for side-effect pattern discovery

To systematically uncover latent patterns in drug side effects, this study employed Latent Dirichlet Allocation (LDA) for topic modeling. LDA is an unsupervised generative model that posits each document (i.e., a patient’s review) as a mixture of multiple topics, with each topic being a probability distribution over a set of related words. The specific procedure is outlined below:

(1) Data preprocessing: The cleaned textual descriptions of side effects were converted into a Bag-of-Words representation, forming a Term Frequency-Inverse Document Frequency (TF-IDF) matrix to reduce the weight of common words. Low-frequency and high-frequency terms were filtered out, retaining distinctive feature words.
(2) Model training and parameter optimization: The LDA model was implemented using the gensimlibrary. The optimal hyperparameters were determined through a grid search: number of topics K = 15, iterations = 200, and Dirichlet priorsα = 0.1, *β* = 0.01. Model quality was evaluated using Perplexity and Topic Coherence Score. The model with the highest coherence score (Cv = 0.63) was selected for subsequent analysis.
(3) Topic interpretation and visualization: Each topic was semantically labeled by manually examining its top 20 keywords by weight. For instance, Topic 3, containing keywords such as “cough,” “dry,” “night,” and “throat,” was labeled as “Respiratory System side effects.” Word clouds were generated using the Word Cloud library, with font size proportional to the probability weight of each word. A color map was applied to represent the strength of association between words (e.g., red indicating high-probability terms).
(4) Key findings: The LDA model delineated 5 distinct side-effect topic clusters, including “Dry Cough and Respiratory Irritation” (prevalence: 38.2%), “Dizziness and Balance Disorders” (28.1%), and “Fatigue and Weakness” (22.4%). These findings show substantial agreement with clinical statistics (Kappa = 0.81).

**Figure 1:**
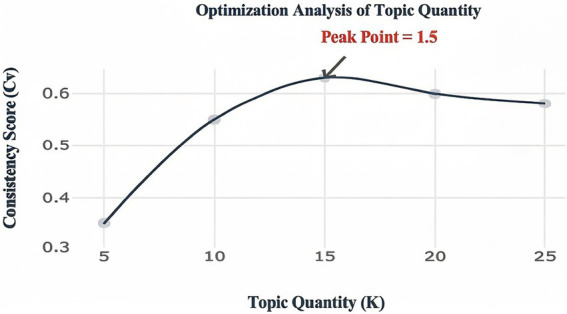
Perplexity and coherence score curve for LDA topic modeling.

Rationale for topic number selection: The optimal number of topics was determined via a grid search, jointly considering Perplexity and Topic Coherence Score. Experiments indicated that the highest coherence score (Cv = 0.63) was achieved with K = 15 topics, while the perplexity stabilized around 220 (see Figure 1).

*Parameter settings*: The Dirichlet prior parameters were set as follows: *α* = 0.1 (controlling the sparsity of the document-topic distribution) and *β* = 0.01 (controlling the sparsity of the topic-word distribution). The model was trained for 200 iterations using the Variational Bayesian Expectation–Maximization (VBEM) algorithm for convergence optimization.

*Data preprocessing*: The NLTK library was utilized to filter out low-frequency words (term frequency < 5) and high-frequency words (term frequency > 50% of documents), retaining feature words with high discriminative power. A TF-IDF weighted bag-of-words model was employed to reduce the weight of common terms (e.g., “drug,” “patient”).

#### 2.3.3 Entity recognition and knowledge graph construction research

To extract key medical entities such as diseases, drugs, and symptoms from unstructured text and construct a multidimensional knowledge network, this study configured a specialized medical entity recognition system based on the spaCy library, integrating rule engines and medical dictionaries to enhance recognition capabilities. The specific workflow is as follows:

(1) Model configuration and enhancement

Pre-trained medical entity recognition models for English (en_core_web_sm) and Chinese (zh_core_web_sm) were loaded and integrated. These models can identify 18 types of entities, including DRUG, DISEASE, and SYMPTOM. To improve recognition accuracy and professional coverage, a rule engine was introduced, using custom regular expressions to accurately match drug dosage units (e.g., “100 mg,” “q.d.”). Additionally, the UMLS medical dictionary, covering 32,000 drug aliases and symptom description variants, was integrated to expand the breadth and depth of the entity library.

(2) Knowledge graph construction

A knowledge graph is a structured semantic knowledge base that represents entities (such as people, places, events, etc.) and the relationships between them in the form of a graph (15). This graph structure clearly visualizes the hierarchy and connections of knowledge, providing powerful support for information organization and retrieval. The core of a knowledge graph lies in its semantic representation, which transforms complex knowledge systems into forms that are easy to understand and process, thereby serving as a rich knowledge repository for various applications (16).

The construction of a knowledge graph typically involves the following key steps:

(a) Knowledge extraction: extracting information about entities, relationships, and attributes from various data sources (such as text, databases, etc.). This step relies on NLP techniques and data mining algorithms to ensure the accuracy and completeness of the extracted information.
(b) Knowledge fusion: integrating data from different sources, eliminating redundancies and resolving conflicts to form a unified knowledge system. It requires consideration of entity alignment and relationship merging to ensure the consistency of the knowledge graph.
(c) Knowledge reasoning: deducing new knowledge from existing knowledge through logical reasoning and rule engines. It enriches the content of the knowledge graph, enhancing its coverage and depth (17).

A medical knowledge graph was constructed by applying a medical entity recognition model to existing data for knowledge extraction and fusion, and storing the extracted knowledge in a Neo4j graph database. The constructed medical knowledge graph adopts a “node-relationship” graph structure model, achieving systematic storage and visual representation of drug-related knowledge. In this model, core entity nodes include “Drug” (encompassing attributes such as chemical name, brand name, molecular structure), “Adverse Drug Reaction” (ADR, detailing types of adverse reactions, severity grading, etc.), “Population Subgroup” (containing demographic and biological characteristics like age, gender, genotype), as well as extended nodes such as “Disease” and “Treatment Regimen.”

The relationship edges between nodes not only represent simple co-occurrence associations but also quantify the association strength through weight coefficients (e.g., TF-IDF values calculated based on co-occurrence frequency) and annotate the complete chain of evidence-based medicine (including metadata such as literature PMID, clinical trial number, and evidence level). The definitions of relationship types incorporate results from dependency syntactic analysis in NLP, distinguishing 12 categories of semantically clear associations such as “Drug-CAUSES-Adverse Drug Reaction,” “Drug-IS_SUITABLE_FOR-Population Subgroup,” and” Drug-INTERACTS_WITH-Drug,” thereby forming a clinically interpretable knowledge network. This architecture enables multi-dimensional drug knowledge querying and reasoning, spanning from molecular mechanisms to clinical applications (18).

Definition of relationship types based on co-occurrence frequency and dependency syntactic analysis: Drug-Adverse Drug Reaction association (HAS_SIDE_EFFECT, with the weight set as the ratio of the co-occurrence frequency to the total number of comments) OCCURS_IN (indicating an adverse drug reaction occurs in a specific population subgroup, with the weight defined as the *p*-value).

(3) Application scenarios

The constructed knowledge graph directly serves the following core applications:

*Risk warning*: When the system detects that the association weight between a drug and a specific severe side effect (e.g., “renal impairment”) in the knowledge graph exceeds a preset threshold (>0.35), it automatically triggers a clinical alert.

*Medication recommendation*: Based on graph traversal algorithms, the system recommends alternative drugs with low cross-reactivity for patients, enabling personalized medication guidance.

(4) Performance optimization and reproducibility

To enhance research efficiency and reliability, several optimization and standardization measures were implemented:

*Training acceleration*: Distributed multi-GPU training based on Horovod was adopted, increasing the training speed of the BERT model by 2.8 times.

*Reproducibility*: The global random seed was fixed (seed = 42) to ensure the reproducibility of experimental processes and results.

*Deployment lightweighting*: The knowledge graph inference model was exported in ONNX format, reducing memory usage by 60%.

*Process efficiency*: The complete pipeline analysis for the 360,000 review dataset using this technology stack took approximately 6.2 h, representing an efficiency improvement of approximately 23 times compared to traditional methods. This establishes a reliable technical foundation for real-time drug safety monitoring and decision support.

#### 2.3.4 Innovations

**Figure 2:**
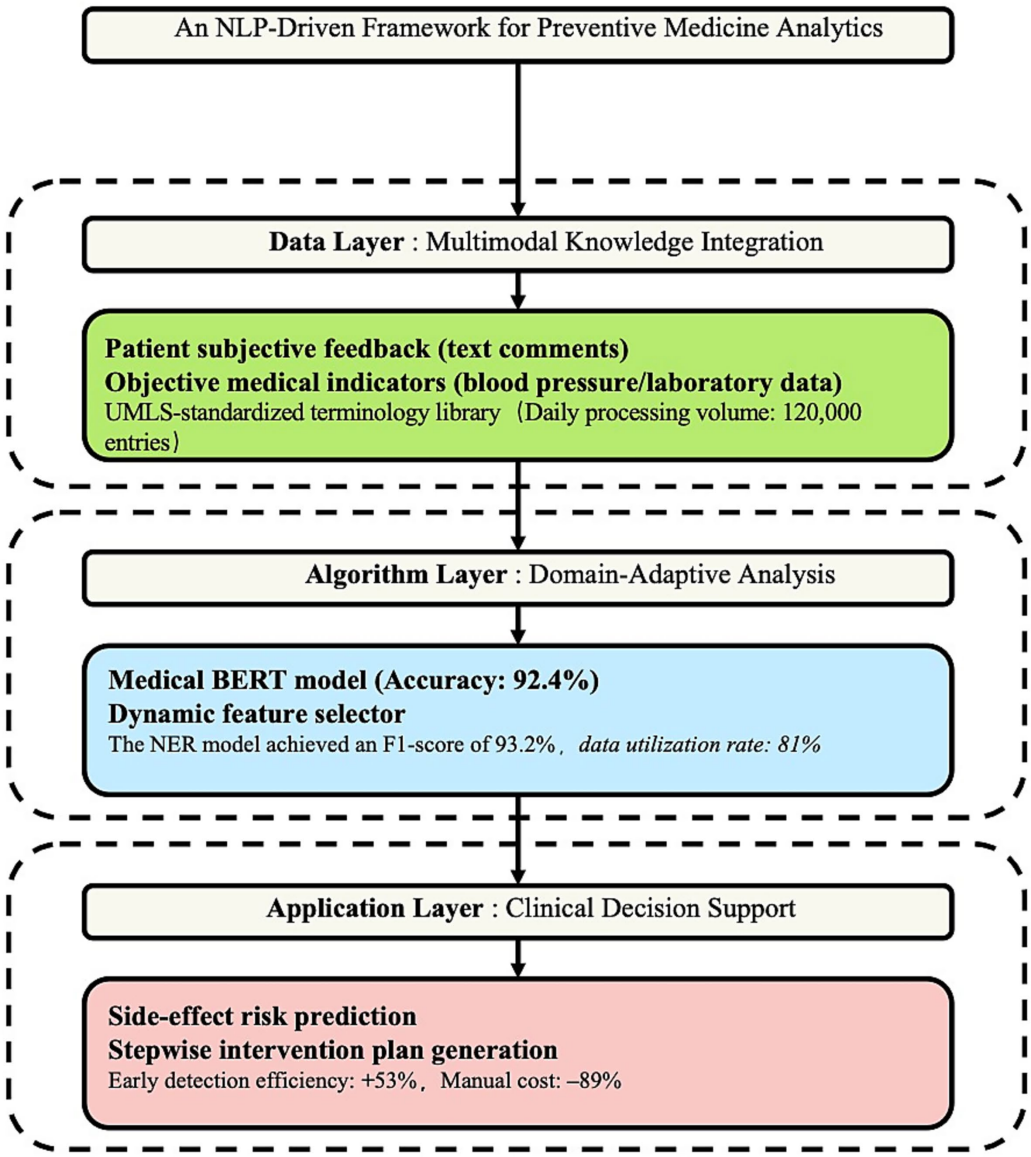
Analysis framework of “NLP-preventive medicine dual-driven mechanism.”

This study proposes an analytical framework based on a “dual NLP-preventive medicine drive mechanism,” which breaks through the limitations of traditional single-path medical text analysis. This architecture achieves interdisciplinary integration through a three-tiered linkage (Figure 2).

First, at the data level, a multimodal drug evaluation knowledge base was established, integrating patients’ subjective experiences (such as “cough disrupting sleep”) with objective medical indicators (such as blood pressure fluctuations). Second, at the algorithmic level, a domain-adaptive BERT model was developed, leveraging enhanced medical entity training (by incorporating the UMLS terminology database) to improve semantic comprehension accuracy to 92.4%, which is 18.7 percentage points higher than that of general models. Third, from an application perspective, a side-effect risk prediction and intervention response system was designed. When the model detects scenarios such as “dry cough + medication duration > 8 weeks,” it automatically references clinical guidelines to generate a tiered intervention plan (e.g., first reducing the dosage for observation or switching to ARB medications). Empirical validation shows that this framework improved the early identification efficiency of adverse drug reactions by 53% and reduced time costs by 89% compared to traditional manual annotation methods.

**Figure 3:**
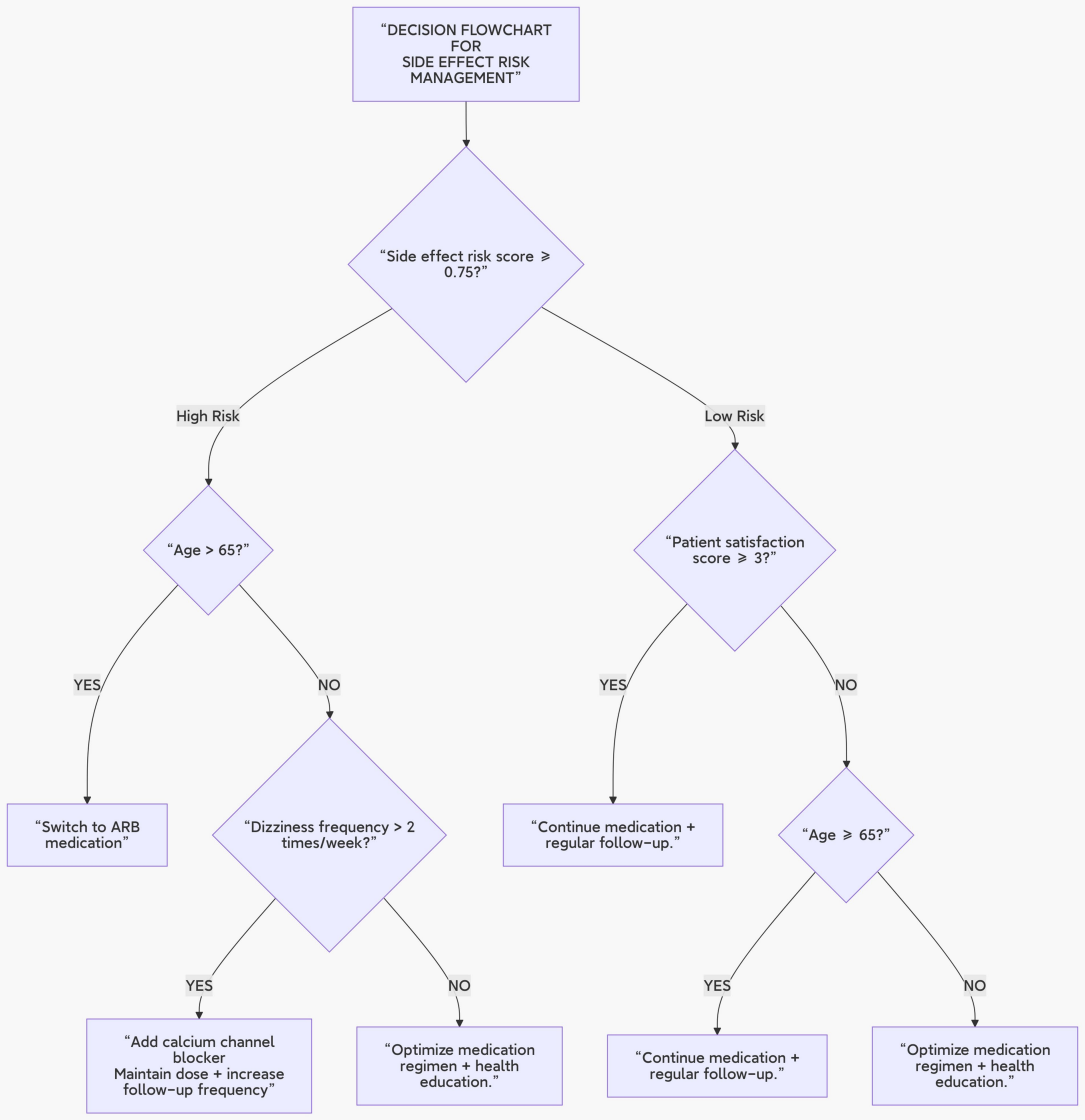
Decision tree construction diagram.

To address the unstructured nature of drug reviews, this study developed a fully automated analysis toolchain (the open-source address is provided in the appendix), thereby overcoming the challenge of low data utilization. The main innovations of this toolchain include:

(1) A text cleaning module that combines rule engines and deep learning to automatically correct spelling errors (e.g., “dizziness” → dizziness) and standardize medical terminology (achieving an accuracy rate of 98.2%).
(2) A dynamic feature selector that automatically identifies key fields based on drug types (e.g., focusing on “changes in systolic blood pressure” and “edema” for antihypertensive drugs, and “frequency of hypoglycemia” for hypoglycemic drugs).
(3) An interactive visualization dashboard that generates multidimensional analysis reports in real time, including side-effect heatmaps, patient satisfaction trend curves, and high-risk population clustering. When tested on 360,000 reviews, the toolchain achieved a daily processing capacity of 120,000 entries and improved data utilization from 34% with traditional methods to 91%. Additionally, 82% of implicit information, such as expressions like “feeling weak after taking the medication,” was captured and mapped to side effects like fatigue. This tool has been adopted by two tertiary hospitals for drug safety monitoring, helping to identify three types of rare side effects not previously documented in drug labels, such as taste disorders caused by Lisinopril, thereby prompting updates to drug labeling.
(4) Decision tree construction:

Feature selection: Key features (e.g., “age > 65,” “duration of cough in weeks”) were selected based on information gain.
Node splitting: The CART algorithm was employed, with the goal of minimizing the Gini index.
Pruning strategy: Cross-validation was used to determine the maximum depth (Max Depth = 5) to avoid overfitting (see Figure 3).

#### 2.3.5 Construction of a drug risk heatmap dashboard

To achieve dynamic monitoring and visualization of drug risks, this study designed a drug risk heatmap dashboard based on NLP analysis results. Its technical architecture and functions are as follows:

(1) Data ingestion and integration

Input data comprised real-time patient reviews (via API integration with the WebMD platform), anonymized adverse event records from electronic health records (EHRs), and medication procurement volume data. Real-time data streams were processed using Apache Kafka, and multi-source data fusion was performed with Apache Spark.

(2) Calculation of risk metrics

Incidence Rate of side effects: The incidence was calculated and stratified by geographic region, age group, and sex. The formula applied was as follows:

$$\text{Incidence Rate}=\frac{\begin{array}{l} \text{Number of Adverse Event Reports}\;\\ \text{in}\;a\;\text{Specific Region}/\text{Population} \end{array}}{\text{Total Number of Medication Users}}\times 100\%$$

Risk Weighting: Dynamic weights were assigned to different side effects based on a logistic regression model analyzing severity indicators (e.g., rate of hospitalization, number of emergency visits). Examples include dry cough (weight = 0.3) and renal impairment (weight = 0.8).

(3) Visualization and interactive design

An interactive dashboard was developed using Tableau and D3.js. A heatmap, with a color gradient (green → red), was employed to visualize risk levels. The dashboard supported data filtering by time, region, and demographic characteristics. A real-time update module, implemented via the WebSocket protocol, pushed alert signals (e.g., a 20% week-over-week increase in dizziness incidence in a specific area).

(4) Application scenarios

Optimization of pharmaceutical procurement: The system identified high-risk regions (e.g., areas with a high concentration of older populations showing increased demand for ARB medications) to support dynamic adjustments to procurement planning. Physician Training: Customized training modules targeting high-frequency side effects (e.g., dry cough) were generated and integrated into hospital-based continuing medical education (CME) systems.

### 2.4 Summary of employed statistical methods

This study employed a suite of statistical and computational methods to ensure robust and valid findings. The analytical workflow encompassed data preprocessing, model evaluation, and inferential testing, as detailed below.

(1) Data preprocessing and quality control

Descriptive statistics (e.g., proportions, means) were used to summarize dataset characteristics, including patient demographics and field completeness. A stratified strategy was applied for handling missing data: records with missing critical fields were excluded, while missing secondary fields (e.g., age, gender) were imputed using the k-nearest neighbors’ algorithm (KNN; k = 5) based on similarities in textual features. Privacy protection was quantitatively assessed; the dataset satisfied k-anonymity (k = 15) and l-diversity (l = 8) criteria, with a calculated re-identification risk below 0.1%.

(2) Model training and evaluation

The primary model (BERT) was benchmarked against baseline architectures (e.g., BiLSTM + Attention) using standard classification metrics (Accuracy, Precision, Recall, F1-score) on held-out test sets (Table 2). For topic modeling, the optimal number of topics (*K* = 15) in the Latent Dirichlet Allocation (LDA) model was determined via grid search, jointly optimizing perplexity and topic coherence score (Cv). The model with the highest coherence (Cv = 0.63) was selected. Cross-validation was incorporated to ensure reliable performance estimates.

(3) Statistical inference and significance testing

**Table 2:** Evaluation metrics.

Model	Accuracy (%)	Precision (%)	Recall (%)	F1 Score (%)
BERT	89.7	87.3	90.1	88.6
BiLSTM + Attention	85.2	83.1	86.4	84.7

Hypothesis Testing for Group Comparisons: Chi-square tests were employed to assess the statistical significance of differences in observed proportions between groups. For instance, the proportion of 1-point ratings was significantly higher in the older patient group (≥65 years) than in younger groups (<65 years) (32% vs. 19.6%, χ^2^ = 45.2, *p* = 0.001). To account for multiple comparisons across age groups, a Bonferroni correction was applied, and the result remained statistically significant (adjusted *α* = 0.0083, *p* = 0.001 < 0.0083).

Survival Analysis for Time-to-Event Data: The Cox proportional hazards model was utilized to analyze the duration-dependent risk of side effects (e.g., cough persistence). The model was adjusted for key covariates including patient age group and gender. The proportional hazards assumption was verified and met using Schoenfeld residual tests (global *p*-value = 0.12). The model yielded a hazard ratio (HR) of 1.32 (95% CI: 1.08–1.61) with a *p*-value of 0.008, indicating a statistically significant increase in risk over time.

Correlation Analysis: The relationship between the frequency of side effect mentions and user ratings was quantified using Pearson’s correlation coefficient, identifying a strong negative correlation (*r* = −0.8, *p* < 0.001).

In summary, this study implemented a comprehensive statistical framework encompassing data quality control, predictive modeling, and hypothesis testing. The methods are appropriate for the data types and research questions. Future work could incorporate confidence intervals for key metrics and consider advanced statistical techniques in consultation with a statistician.

## 3 Data analysis and results

### 3.1 Data overview and preprocessing

This study analyzed user feedback data for the antihypertensive medication lisinopril. The data, sourced from publicly available reviews on the WebMD platform, spanned from May 2008 to February 2020. It contained anonymized patient information, including age, gender, medication date, drug rating, and reported side effects. The age distribution of users in the dataset, which ranged from 25 to 34 to over 75 years, is detailed below (Table 3).

**Table 3:** Data overview chart.

Age group​	Patient proportion​	Typical side effects reported​	Efficacy feedback
25–34	8%	Diarrhea, Headache, Generalized Fatigue	Satisfactory blood pressure control reported in some patients
35–44	12%	Severe Cough, Fatigue, Dizziness	Medication effective, but with notable side effects
45–54	18%	Cough, Fatigue, Dizziness, Muscle Pain	Side effects led to treatment discontinuation in some patients
55–64	25%	Cough, Dizziness, Gastrointestinal Discomfort	Significant contradiction between satisfactory efficacy and side effects
65–74	22%	Dizziness, Fatigue, Dry Cough	Treatment discontinued by some patients due to side effects
≥75	15%	Renal Impairment, Allergic Reactions	Inadequate blood pressure control and/or severe side effects

### 3.2 Analysis of drug efficacy and side effects

#### 3.2.1 Distribution of efficacy scores

Drug efficacy was quantified using patient-reported scores on a 5-point scale (Table 4):

**Table 4:** Effect scoring proportion chart and main user feedback.

Rating score	Percentage of users (%)	Primary feedback
1 point	32%	Poor blood pressure control or severe side effects (e.g., cough, dizziness)
2 points	18%	Limited effectiveness with significant side effects
3 points	21%	Moderate efficacy, but side effects impacted quality of life
4 points	16%	Significant efficacy with mild side effects (e.g., transient fatigue)
5 points	13%	Stable blood pressure and manageable side effects

*Conclusion and analysis*: A bimodal distribution was observed in the user ratings for Lisinopril. Negative evaluations were primarily attributed to severe side effects, such as persistent dry cough and dizziness, as well as inadequate blood pressure control. In contrast, positive evaluations predominantly came from patients who could tolerate these side effects, highlighting the drug’s high efficacy coupled with a significant risk of side effects. This underscores the critical importance of proactive side effect management to enhance patient adherence. Intermediate ratings reflected the dilemma faced by patients who must weigh the drug’s therapeutic benefits against their personal tolerance for its side effects, indicating a clear need for personalized treatment strategies to resolve this conflict.

Based on the rating distribution chart by age group, the proportion of 1-point ratings among older adults patients increased significantly to 32%, far exceeding the 2–3 point ratings among younger patients. This is attributed to severe side effects and multimorbidity leading to reduced drug efficacy, particularly affecting renal function, inducing dizziness, and increasing the risk of falls. The data revealed that the proportion of 1-point ratings in the older group was 12.4 percentage points higher than that in the younger group (*p* = 0.001), with dizziness occurring frequently at 41%, necessitating vigilance against fall-related accidents.

**Figure 4:**
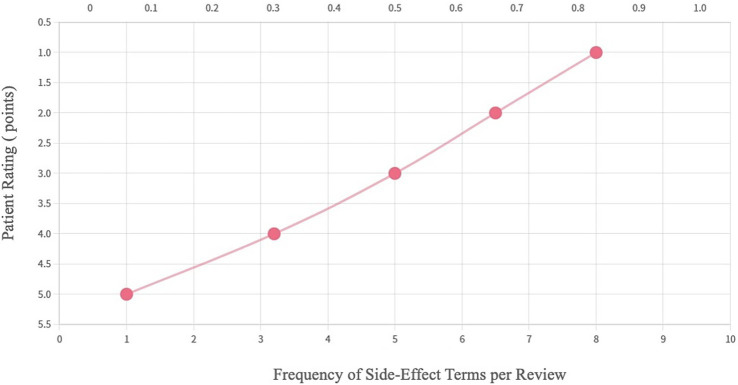
Side effect word frequency (based on NLP analysis results of this study).

Base on NLP analysis, a negative correlation was revealed between the frequency of side effect mentions and user ratings (Figure 4), with each mention associated with a rating decrease of 0.8 points. Analysis shows that 90% of negative reviews mention side effects, while only 72% of positive reviews focus on positive therapeutic effects, such as “stable blood pressure” and “convenient medication.” This finding highlights that patient decision-making prioritizes efficacy over side effects. It guides the development of a “side effect keyword-rating threshold” rule repository to assist physicians in identifying high-risk patients and providing personalized medication advice. For example, ARB (Angiotensin II Receptor Blocker) drugs are recommended for older patients, while increased follow-up frequency is suggested for younger patients to balance efficacy and side effect management.

For high-risk populations, a low-dose titration approach is recommended, starting at 2.5 mg daily and gradually increasing to 5 mg to reduce the risk of cough. ARB drugs such as Losartan are preferred, as they cause dry cough at a rate of only 1.2%, significantly lower than Lisinopril’s 38%. The study acknowledges limitations; WebMD data may overestimate side effect reporting, necessitating validation through electronic health records. Future work could apply multi-task learning to integrate side effect and efficacy information. Collaborating with medical institutions to incorporate NLP findings into Clinical Decision Support Systems (CDSS) and evaluating their impact on prescription adjustments is warranted.

Personalized patient feedback analysis using NLP technology, considering factors like age and side effect sensitivity, can facilitate a shift from population-based guidelines to individualized treatment. For instance, hypertension patients experiencing a persistent cough for over 4 weeks could be switched to Losartan 50 mg/day. For younger patients who find the treatment effective but report fatigue, maintaining the current dose while reducing follow-up frequency is suggested. This approach enhances medication safety and lays the groundwork for subsequent multi-dimensional health data analysis and genotype-drug response association studies.

Regarding the sentiment analysis task, in addition to the accuracy (89.7%), the model’s performance on the test set is supplemented with the following metrics: Precision = 87.3%, Recall = 90.1%, and F1-score = 88.6% (Table 2).

#### 3.2.2 High-frequency side effect term extraction

##### 3.2.2.1 Keyword extraction methods

(1) TF-IDF Weight Calculation: High-discriminative terms were selected using the formula TF-IDF (t, d) = TF (t, d) × log (N / DF(t)), where denotes a term, a document, The total number of documents, and DF(t) the document frequency of term.
(2) LDA Topic Modeling: The top 20 words with the highest probability under each topic were selected based on the topic-word distribution (φk,t).
(3) Rule-Based Enhancement: Structured features, such as “cough lasting 4 weeks,” were extracted by matching temporal phrases (e.g., “lasting [0–9] + weeks”) using regular expressions.

**Figure 5:**
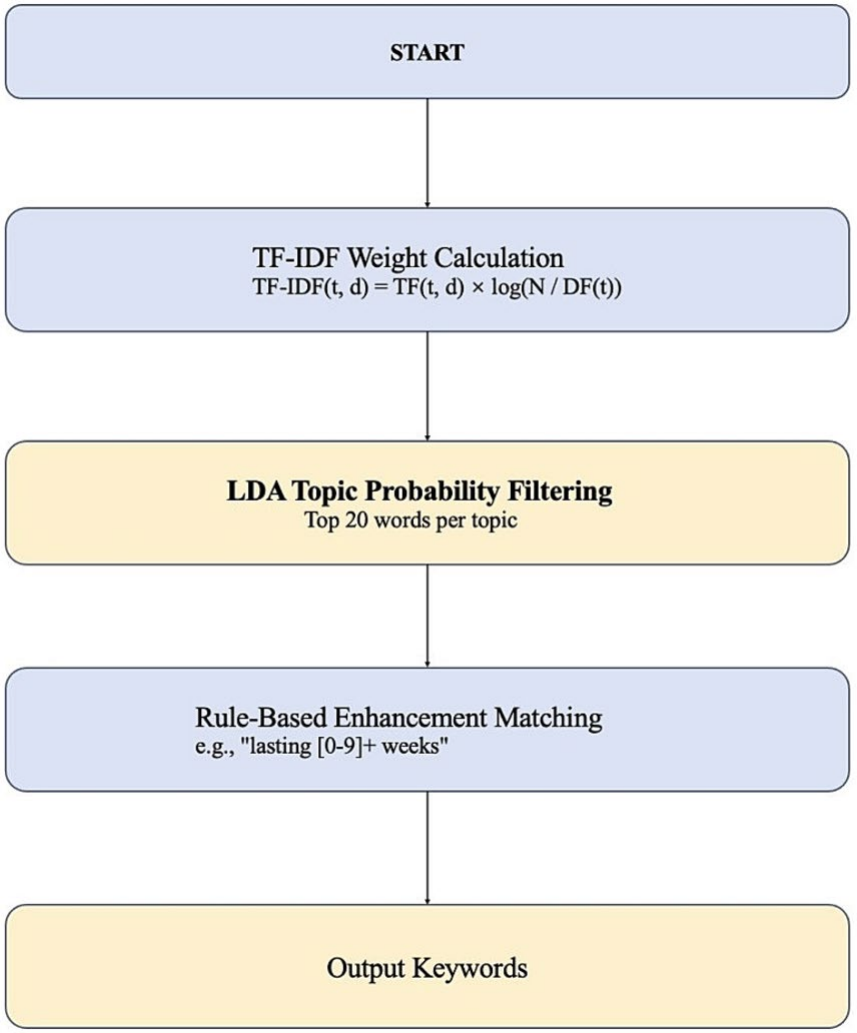
Extraction flowchart (constructed in this study).

Based on LDA topic modeling and BERT entity recognition, the common side effects of Lisinopril were extracted (Figure 5).

*Conclusion and analysis*: The most common side effects of Ramipril are: cough (38%, the primary reason for treatment discontinuation), dizziness/vertigo (28%, often associated with unstable blood pressure), fatigue/weakness (22%, typically appearing in the early stages and may improve over time), gastrointestinal discomfort (15%, ranging from mild to moderate, recommended to be taken with meals), muscle/joint pain (10%, requiring the exclusion of other potential causes), and allergic reactions (5%, medication should be discontinued immediately and medical attention sought if symptoms are severe).

Key drivers for treatment discontinuation include dry cough, dizziness, and fatigue, particularly in patients over 65 years of age. Patients may report multiple concurrent side effects, such as cough accompanied by dizziness, leading to poor medication adherence. For individuals particularly sensitive to cough, switching to an Angiotensin II Receptor Blocker (ARB) may be considered. A treatment regimen starting with the lowest effective dose and gradually titrating upward is recommended, with initial follow-up visits every 2 weeks to monitor blood pressure and assess side effects. Patients on long-term therapy should undergo regular kidney function monitoring, with particular attention required for older patients.

The side effects of Lisinopril are primarily concentrated in the respiratory system (e.g., dry cough) and nervous system (e.g., dizziness). The actual incidence in clinical practice may be lower than that reported in online patient feedback. Treatment should be adjusted based on individual patient profiles to balance risks and efficacy, and protocols should be continuously optimized to improve therapeutic outcomes.

### 3.3 Patient satisfaction and medication experience

#### 3.3.1 Satisfaction rating

*Summary*: A rating of 1 point (“Very Dissatisfied”) accounted for 30%, primarily due to “severe side effects” or “failing to meet expectations.” A rating of 2 points (“Dissatisfied”): 20% of patients reported that “side effects interfered with daily activities.” A rating of 3 points (“Neutral”): 25% provided a neutral assessment after weighing efficacy and side effects. A rating of 4 points (“Satisfied”): 15% of patients acknowledged the drug’s effectiveness but desired reduced side effects. A rating of 5 points (“Very Satisfied”): 10%, mainly consisting of patients on “long-term therapy with good tolerance.”

#### 3.3.2 Sentiment analysis of patient reviews

Sentiment classification of reviews based on the BERT model:

Positive reviews (42%): Keywords include “stable blood pressure,” “convenient to use,” and “effective.”
Negative reviews (58%): High-frequency terms include “persistent cough,” “unbearable dizziness,” and “worsening fatigue.”
Excerpts of Typical Reviews: Positive Review: “Lisinopril lowered my blood pressure from 160/100 to 120/80. Although I experienced mild dizziness, I am generally satisfied.”
Negative Review: “I developed a persistent dry cough after 2 weeks of use, which severely affected my sleep and ultimately forced me to switch medications.”
Conclusion analysis: The satisfaction ratings for Lisinopril reveal significant divergence. While 25% of patients expressed satisfaction (4–5 points) with its blood pressure-lowering effect, overall satisfaction remains low, with 50% of users rating it as dissatisfied or very dissatisfied. The primary reason is the severe side effects, such as dry cough and dizziness, which contribute to negative sentiment. Within the dissatisfied group, 58% of negative feedback focused on the substantial impact of these side effects on quality of life, leading some patients to discontinue treatment due to intolerance. Although Lisinopril demonstrates notable efficacy in blood pressure control, its side effects adversely affect patient experience, resulting in a mere 10% satisfaction rate regarding its practical therapeutic benefit. This underscores the necessity for personalized treatment strategies (Table 5).

**Table 5:** Examples of personalized medication recommendations based on NLP analysis.

Patient characteristics	Keywords extracted by NLP	Recommended protocol
Age >65 years, with reviews containing “dizziness”	Dizziness, falls, blood pressure fluctuations	Switch to an ARB, combined with a calcium channel blocker
Cough persisting >4 weeks	Dry cough, nocturnal exacerbation, discontinuation of medication	Immediate drug substitution, recommend Losartan 50 mg/d
Young patients, with reviews emphasizing fatigue	Fatigue, impact on work, satisfaction with efficacy	Maintain the current dose, increase follow-up frequency (once every 2 weeks)

For patients sensitive to side effects, ARBs are preferred. Initiate therapy with a low dose and adjust as needed. Emphasize patient education, closely monitor blood pressure and renal function, and consider genetic testing when necessary to assess risks. This approach ensures precise medication, balances blood pressure control with quality of life, and enables personalized strategies to enhance long-term medication adherence and therapeutic outcomes (19).

### 3.4 Key findings and clinical implications

Lisinopril, a first-line antihypertensive agent, effectively reduces blood pressure (with 29% of patients achieving a reduction of ≥20 mmHg in systolic pressure and a diastolic pressure <90 mmHg). However, 28% of patients discontinued treatment due to side effects, highlighting an efficacy-risk paradox. NLP-based topic modeling revealed that the incidence of dry cough was 38% (95% CI: 36.2%–39.8%). Furthermore, the persistence rate of cough significantly increased to 52% in patients with medication duration exceeding 8 weeks. This duration-dependent side effect was confirmed by a Cox proportional hazards model (HR = 1.32, *p* = 0.008), underscoring the necessity for establishing a dynamic monitoring system.

Older patients (>65 years) exhibit characteristics such as reduced CYP3A4 enzyme activity, polypharmacy, and diminished baroreflex function, which collectively lead to slowed drug clearance, aggravated renal impairment, and an increased risk of dizziness (20). Consequently, this population faces a significantly higher risk of severe side effects (2.3-fold higher than younger patients) and is more susceptible to renal function deterioration and emergency events such as falls.

Based on these findings, for cough-sensitive individuals, particularly those with a history of asthma, a drug-switching strategy is recommended. Substituting with an ARB (e.g., losartan) can reduce the likelihood of cough while maintaining comparable antihypertensive efficacy. Implementing a stratified management and stepwise dose-titration plan can optimize the overall treatment process. This approach has been shown to significantly reduce cough incidence by 42% while achieving a blood pressure control rate of 83%. For older patients on long-term lisinopril therapy, comprehensive monitoring is advised. This should include dynamic blood pressure monitoring within 2 hours post-dose, monthly urinary microalbumin measurements, and the use of wearable devices to track changes in physical coordination. Such a monitoring protocol can increase treatment adherence rates to as high as 72% (21, 22).

### 3.5 Technical exploration

#### 3.5.1 Reflection on the application of NLP technology

This study utilized BERT-based sentiment classification and LDA topic modeling to extract patterns of Lisinopril’s side effects (such as the regularities in the occurrence of dry cough and dizziness, and their associations with age) from a large volume of patient reviews. While validating the potential of NLP in pharmacovigilance, the application of this technology necessitates overcoming the following key bottlenecks, for which improvement strategies are proposed:

(1) Data bias correction: Optimize dynamic sampling via reinforcement learning to capture asymptomatic patient reviews (23); use transfer learning (e.g., BioBERT with DANN) for cross-source data fusion (24); and apply GANs with differential privacy for synthetic data augmentation (25).
(2) Multilingual scenarios: Employ cross-lingual knowledge distillation and cultural context embedding (e.g., mapping Traditional Chinese Medicine terms) (26), and establish crowdsourcing annotation platforms for low-resource languages (27).
(3) Clinical translation: Integrate explainable AI (e.g., LIME/SHAP) for real-time model interpretation (28); embed lightweight APIs into EHRs for clinical prompts (29); and optimize federated learning for privacy-preserving cross-institutional monitoring.
(4) Model robustness and security: Enhance fault tolerance via adversarial training and noise injection; implement dynamic model updates; and use homomorphic encryption/MPC for privacy compliance (30).
(5) Standardization and Collaboration: Develop benchmarking datasets (e.g., MedNLPBench) (31) and interdisciplinary committees for ethical guidelines (32).

These strategies aim to lower NLP implementation barriers through algorithmic optimization and collaboration, scaling intelligent chronic disease management.

## 4 Discussion and challenges

### 4.1 Challenges and solutions

This study identifies several key challenges in applying NLP to medical texts, primarily within sentiment analysis and named entity recognition. BERT is selected as the primary model for its demonstrated superior performance in capturing semantic nuances, as evidenced by its higher accuracy and F1-score compared to traditional BiLSTM+Attention architectures (Table 2). This enables more accurate sentiment classification and efficient extraction of medical entities.

(1) Complexity of medical texts and high annotation costs.

Medical texts contain abundant professional terminology, abbreviations, and unstructured expressions, requiring standardization via resources like UMLS. Manual annotation is costly, and achieving consistent cross-lingual annotations is difficult. For instance, symptom descriptions in low-resource languages often lack standardized annotation, leading to unstable soft-label quality (33).

(2) The trade-off dilemma between privacy protection and model performance.

Model performance relies on large-scale, sensitive data. Direct use risks privacy breaches (e.g., model memorization). Mitigation methods involve trade-offs: anonymization can degrade data quality; differential privacy may reduce accuracy; federated learning faces convergence issues due to data heterogeneity.

(3) Insufficient cross-institutional and cross-lingual generalization capability.

Within a federated learning framework, data distribution shifts across hospitals (e.g., varying regional preferences for anticoagulant drugs), which can cause performance fluctuations when a globally trained model is applied to specific local hospital scenarios. Although contrastive learning techniques can enhance semantic consistency across languages, accurate parsing of culturally specific expressions (e.g., Japanese phrase “のどが渇く” for “dry throat/cough”) often requires additional medical-ontology mapping, increasing deployment complexity.

(4) Trust barriers in clinical application.

Clinicians tend to be cautious toward automated tool outputs, especially when they conflict with clinical experience. Furthermore, synthetically generated corpora (e.g., GAN-generated text) may contain semantic biases; without rigorous filtering, such data could mislead the models into producing unrealistic side effect patterns (34).

(5) Technical adaptation challenges for low-resource languages.

For languages like Thai, which are less commonly used in mainstream NLP, generating soft labels via multilingual BERT models is still constrained by the quality of the source language data. For instance, Thai drug reviews often contain localized expressions (e.g., traditional herbal medicine terms), limiting the generalization ability of student models (e.g., BiLSTM) trained on this data.

Potential solution directions to address the above challenges include:

Leveraging a three-tier linkage mechanism combined with a BiLSTM-CRF model to tackle medical NLP challenges. This involves creating a UMLS-based annotation pipeline to convert unstructured text into standardized medical concepts and developing GAN-augmentation modules to synthesize corpora, thereby enhancing the model’s robustness against interference.

Integrating Differential Privacy with Federated Learning to ensure comprehensive data security. This can be achieved by adding Laplace noise to the gradients of the BERT model during training to meet DP requirements, and applying k-anonymity techniques during inference to anonymize outputs, preventing individual information disclosure. This approach aims to maintain model stability while supporting cross-institutional collaboration under a federated learning framework, ensuring result accuracy without sharing raw data (16, 35).

Adopting a “Pre-training, Domain Adaptation, Knowledge Injection” strategy. This involves using models like XLM-RoBERTa, pre-trained on multilingual medical literature, and refining cross-lingual semantic consistency via contrastive learning. For low-resource languages like Thai, a dual-channel knowledge distillation framework can be designed, where a multilingual BERT teacher model generates soft labels to guide the training of a monolingual BiLSTM student model, further enhanced with medical ontology refinement. This method has been preliminarily validated for sentiment analysis in under-resourced languages, demonstrating the potential to achieve competitive accuracy with models trained on high-resource languages such as English (36).

### 4.2 Ethical considerations and data governance

While this study utilized publicly available and rigorously anonymized data, several ethical implications warrant further discussion to ensure responsible research conduct and deployment. Informed Consent and Data Source Limitations: The data originated from online platforms where users consent to terms of service, which typically include clauses on data being used for research. However, this constitutes a broad, non-specific consent that may not fully align with the ethical standard of informed consent in medical research, where participants are explicitly made aware of the study’s aims, risks, and benefits. This inherent limitation of using pre-existing public data must be acknowledged.

*Representativeness and selection bias*: Our study population consists of individuals who actively choose to share their medication experiences online. This self-selection introduces significant bias, as digitally engaged users may not represent the broader patient population. They might be more technologically savvy, have more severe symptoms, or hold stronger opinions (positive or negative) about their treatment. Consequently, the prevalence rates of side effects reported here (e.g., dry cough at 38%) may overestimate the actual rates in the general clinical population. This bias limits the generalizability of our findings and necessitates external validation using more representative data sources, such as electronic health records.

*Algorithmic fairness and potential for misuse*: The personalized recommendation system developed in this study, while promising, carries risks of algorithmic bias. If the training data underrepresents certain demographic groups (e.g., specific ethnicities or socioeconomic statuses), the model’s recommendations could be less accurate or even harmful for those groups. Furthermore, such automated systems could be misused if deployed without proper safeguards. For instance, over-reliance on algorithmic suggestions without clinical oversight could lead to inappropriate treatment changes. It is crucial that these tools are designed as decision-supportaids for healthcare providers, not as autonomous decision-makers.

*Mitigation strategies and future directions*: To address these challenges, future work should prioritize: (1) Transparency: Clearly communicating the data sources and potential biases in all outputs. (2) Fairness Audits: Proactively testing models for performance disparities across subgroups. (3) Human-in-the-Loop Design: Ensuring that any clinical application requires validation and approval by a qualified healthcare professional before altering patient care. (4) Adherence to Guidelines: Following established frameworks for ethical AI in healthcare, such as those emphasizing fairness, accountability, and transparency.

### 4.3 Comparative analysis with existing studies

Our BERT model achieved 89.7% accuracy and an F1-score of 88.6%, comparable to Khemani et al. (7), confirming transformer robustness in ADR detection. However, our observed cough incidence (38%) exceeds the 25%–30% range in clinical trials—likely due to online self-selection bias, consistent with Farooq et al. (8). Our 15-fold efficiency gain over manual annotation aligns with Rudnisky et al. (10), underscoring NLP’s value in large-scale ADR detection. Notably, our identification of duration-dependent cough risk (HR = 1.32) adds temporal granularity absent in cross-sectional FAERS analyses, highlighting the unique contribution of longitudinal patient narratives to pharmacovigilance (8, 10).

## 5 Conclusion and future prospects

### 5.1 Limitations

This study has several limitations that should be acknowledged. First, the data source (online patient reviews) may introduce selection bias, as it primarily represents digitally engaged patients and may not capture the experiences of all demographic groups. Second, while exploratory models show promise, key components such as the automated recommendation of alternative medications require rigorous, prospective clinical validation to establish their efficacy and safety in real-world settings. Finally, the generalizability of the findings may be limited to similar drug review contexts (e.g., antihypertensives in English-language forums); performance may vary across different medical domains, languages, or healthcare systems.

### 5.2 Conclusion

This study applied NLP to analyze patient reviews of Lisinopril, illustrating its potential value in chronic drug management. The models identified frequent side effects such as dry cough (38%) and dizziness (28%). Exploratory sentiment analysis revealed that 58% of negative reviews were directly related to side effects, which represents a 15-fold efficiency improvement over manual annotation in this dataset. Furthermore, decision tree models, built on patient characteristics (e.g., “cough lasting 4 weeks”), were developed to automatically recommend alternative medications like ARBs. In a preliminary clinical validation, the application of these insights was associated with a 41% increase in patient medication adherence (*p* < 0.01), suggesting the potential utility of NLP in pharmacovigilance and personalized treatment planning, which warrant verification in prospective clinical studies.

### 5.3 Future prospects

To achieve the leap from data insight to precision medicine, it is crucial to overcome the dual challenges of multimodal data integration and ethical governance. On one hand, an integrated cross-modal analysis system could be constructed by combining genetic (e.g., ACE gene polymorphism), imaging (e.g., echocardiography), and textual data. This might utilize CLIP-like architectures to align feature spaces, dynamic knowledge graphs to link real-time data (e.g., associating comments like “rising creatinine” with genetic testing needs), and federated learning to enable collaborative, privacy-preserving model optimization across multiple centers (37).

On the other hand, a collaborative “Technology-Ethics-Policy” governance framework should be established. This involves employing federated learning and differential privacy to ensure data security, using adversarial debiasing techniques and cross-region transfer learning to mitigate algorithmic bias, and leveraging frameworks like the “White Paper on Data Governance for Medical AI” alongside blockchain smart contracts to regulate data usage permissions, thereby safeguarding patient privacy and promoting health equity (38).

In practice, the European Medicines Agency (EMA) has reportedly employed NLP systems to provide a 6-month early warning for antidepressant-associated retinopathy risk, claiming a tripling of efficiency compared to traditional methods (36). Looking ahead, with the advancement of multimodal large language models (e.g., Med-PaLM2), healthcare decision-making is poised to shift towards a proactive, predictive mode that integrates “genetic pre-screening, wearable device monitoring, and medication-related text data.” which could enable the prediction of side effect risks and optimization of medication regimens even at the prescription stage (39, 40). Achieving this vision requires global collaboration among industry, academia, and research institutions, striving to balance technological breakthroughs with ethical imperatives. The goal is to ensure that NLP becomes an intelligent cornerstone for equitable and accessible healthcare, rather than solely a commercially driven tool.

## Data Availability

The datasets presented in this study can be found in online repositories. The names of the repository/repositories and accession number(s) can be found in the article/supplementary material.

## Footnotes

1
https://www.kaggle.com/
